# Proactive Schema Based Link Lifetime Estimation and Connectivity Ratio

**DOI:** 10.1155/2014/172014

**Published:** 2014-01-09

**Authors:** Bouamoud Bachir, Ouacha Ali, Habbani Ahmed, Elkoutbi Mohamed

**Affiliations:** ^1^SIME Lab, MIS Team, ENSIAS, University of Mohammed V SOUISSI, Madinat Al Irfane, BP 713, Agdal, Rabat, Morocco; ^2^LEC Lab, MIS Team, EMI, University of Mohammed V AGDAL, Rabat, Morocco

## Abstract

The radio link between a pair of wireless nodes is affected by a set of random factors such as transmission range, node mobility, and environment conditions. The properties of such radio links are continually experienced when nodes status balances between being reachable and being unreachable; thereby on completion of each experience the statistical distribution of link lifetime is updated. This aspect is emphasized in mobile ad hoc network especially when it is deployed in some fields that require intelligent processing of data information such as aerospace domain.

## 1. Introduction

Ad hoc networks have given numerous innovative possibilities in using computer networks in several situations. In fact mobile ad hoc networks are based on wireless communication; for that reason the requirement of many of services developed for wired networks does not match with resource availability in mobile networks.

In Manet, nodes forming a mobile network are not joined to any infrastructure and form networks on the fly during various time spans. When forming a mobile network there are many factors of complexity such as the shared wireless medium, the mobility of the nodes, the energy constraints, and the agitation of nodes that may join or leave the carrier range anytime during the network lifetime.

In context of high mobility, the routes in the network usually have a short life span. A route may or may not exist for the entire duration of a data communication session. Due to the dynamic changes of the factors that affect the performance in a mobile ad hoc network, it would be convenient that any proposed optimizations should consider this dynamics that acts on nodes and links which interconnect them. In this context, knowledge of network must also have the same character in terms of taking into account factors such as available bandwidth, delays, and the lifetime of nodes in the process of selection of multipoint relays.

In proactive routing mechanism, the use of relays aims to reduce the broadcast messages senders and then the number of flooded messages; here we highlight the importance of relay in OLSR protocol case, since they become the only responsible of broadcasting topology control messages. While this approach is pleasing to the eye, besides some control functions that are necessary to prevent an eternal duplication of broadcast messages, it is required to select relays in reliable manner; indeed defects in the reception of broadcast packets from MPR nodes can greatly affect the rate of delivery of packets across the network.

Thus all applications in ad hoc network depend on reliable and efficient routing of packets. Hence, it is extremely important to design routing protocols that can work within several constraints particularly those of aerospace applications, namely, mobility and lack of energy resources, and provide support for all higher level applications.

There have been many existing routing protocols for ad hoc networks emphasizing different implementation scenarios. However, the basic goals have always been to devise a routing protocol that minimizes control overhead, packet loss ratio, and energy usage while maximizing the throughput. Because these types of network can be used in a variety of situations (disaster recovery, battlefields, conferences, and so on), they differ in terms of their requirements and complexities.

The key idea of this paper is to apply our link reliability estimation model to the multipoint relays selection and routing calculation of the standard OLSR protocol in order to guarantee reliable data transmission. In order to judge the reliability of the links in our link reliability estimation model, we use a statistic inference method that infers the reliability of links by accumulated past information from experimental results. That is, reliability of the links can be estimated by accumulated past information of link lifetime value.

There are two contributions in this paper. First one is the establishment of a link lifetime model to estimate the reliability of links based on a statistical distribution, which is constructed by the value that a given node observes. Second one is the introduction of the later model to optimize the selection of multipoint relays in the context of a proactive routing.

The paper is organized as follows. In Section 2, we present some previous works whose aim is to improve routing reliability based on efficient MPR selection. In Section 3, we introduce a new composite metric based on connectivity ratio and link lifetime prediction.  Section 4 contains a set of simulations and results which concern the evaluation of our proposal. In Section 5, we evaluate the performance of the proposed scheme. Lastly in Section 6 we conclude and discuss future works.

## 2. Related Work

In the context of Proactive routing, the multipoint relay techniques perform very well to desseminate the broadcasted packet into the network. Indeed, many works present in the literature [1–8] were interested in this topic. This puts in evidence that multipoint relaying schemes still arouse the researchers interest.

In fact multipoint relay (MPR) nodes play an important role to deliver broadcast packets to their next MPR nodes as well as their neighbour nodes; failure to receive broadcast packets from MPR nodes can greatly affect the network performance. Then selecting reliable MPR based on link lifetime and ratio of connectivity present promising issues to improve routing performances

On one hand, reliability is a major issue in routing and some previous studies [9, 10] focused on it such that the shortest paths are usually preferred to route packets. Such as we assume the shortest path may fail quickly, because some of wireless links on the shortest path may be broken shortly after the establishment of the path, due to velocity and direction of nodes.

The velocity of the mobile nodes within a MANET is not unchanging. At the same time as there is no speed control of the wireless devices a desirable protocol for MANET should perform well both in relatively static and in totally vibrant network circumstances. The direction of a node's mobility is not known in advance. One common incident is that a node travels to a way where the density of nodes is low or there is no neighbor node in the carrier range.

Abundant research contributions to wireless routing are based on online nodes measurements in order to categorize nodes or links which are preferentially used in the established paths whereas their common weakness is inability to prevent possible change in links status occurring in future. Indeed link qualified as reliable based on past or current measurements may become unreliable with time because of dynamic nature of mobile environments. These drawbacks conducted others in [11, 12], to investigate some prediction method to expect the link availability in a continuous manner during a time span, based on link lifetime.

The residual link lifetime estimation is based on an offline generalized link lifetime distribution, which is unavailable in runtime. However, knowing that the link lifetime distribution is approximately a normal distribution, the trend of residual link lifetime changes can be deduced in advance.

Since the environment conditions of network are changing and velocity and direction of node are not known in advance, the parameters of links lifetime distribution are significantly correlated with the latter assumption.

## 3. Metric Proposal

### 3.1. Link Lifetime

Beginning from a typical example of a mobile ad hoc network consisting of a set of nodes among which a dynamic establishment of links such as *G*(*V*, *E*) is a direct graph and *V* is the set nodes and *E* is the set of links *l* = (*x*, *y*), where the node *y* is within the transmission range of *x*. In general cases routing metrics have attributed values which we call weights associated with each edge:

 hop(*x*, *y*): number of hops between node *x* and node *y*.
 
*N*_1_(*x*) = {*y* | hop(*x*, *y*) = 1}, set of 1-hop adjacent nodes.
 
*N*_2_(*x*) = {*y* | hop(*x*, *y*) = 2}, set of 2-hop adjacent nodes.

If we focus on the link state change, it varies according to the distance between a pair of nodes such as whether they are in the career range of each other. From statistical point of view this network could be assimilated into a statistical population of nodes.

**Figure 1:**
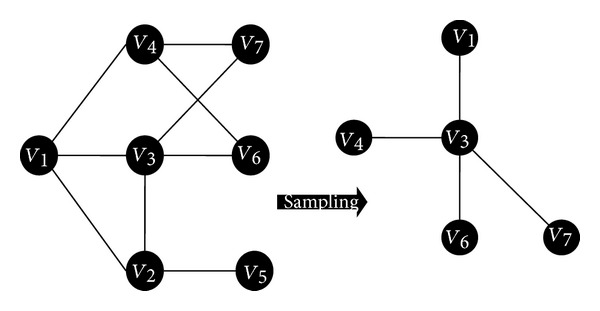
Typical example of nodes sampling.

Each node in the network, for example, (Figure 1) has a changing set of *N*_1_(*x*) that can be considered as a random and repeated sample of the population, each time using various sample size *n*. From each of these repeated samples we calculate independent sample means to form a sampling distribution mean.

Our concern in the current article is to seek for link lifetime measurement method based on what each node observes on the sample whose size is |*N*_1_(*x*)|. The distribution of link lifetime was the concern of many research works such as [13] where the authors illustrate that links lifetime has a normal distribution.

The main works present in the recent literature interested to link lifetime prediction are based on an offline generalized link lifetime distribution, which is unavailable in runtime. The principal weakness of this approach is being biased; in other term it is dependent on the environment conditions which affect the latter distribution parameters such as its mean *m* and variance *σ* ^2^; then the only way to be efficient is by experiencing the network and generating the distribution to know its parameter on which the routing algorithms are based. So the main idea of our proposal is to use the results obtained from samples of link lifetime to describe the population and process this statistics to estimate the parameters of the latter distribution dynamically.

**Figure 2:**
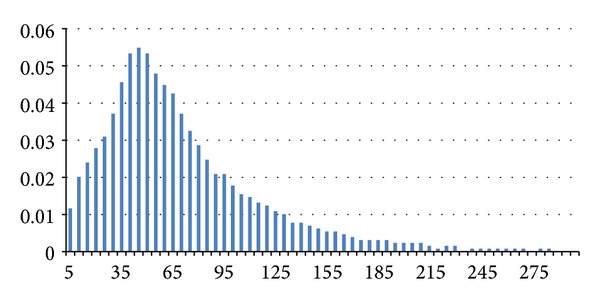
Distribution of link durations in a Gauss-Markov scenario.

**Figure 3:**
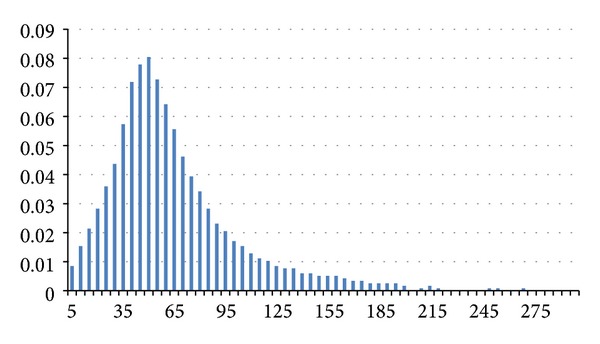
Distribution of link durations in a Random Waypoint scenario.

**Figure 4:**
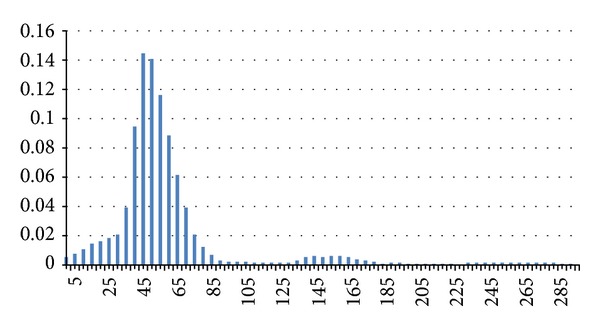
Distribution of link durations in a Manhattan Grid scenario.

Hereafter we present some interesting results that appeared in [13]; Figures 2, 3, and 4 illustrate the distribution of link lifetime in several mobility scenarios, namely, Gauss-Markov, Random Waypoint, and Manhattan Grid. The main feature of this graph is that it has almost a normal distribution.

**Figure 5:**
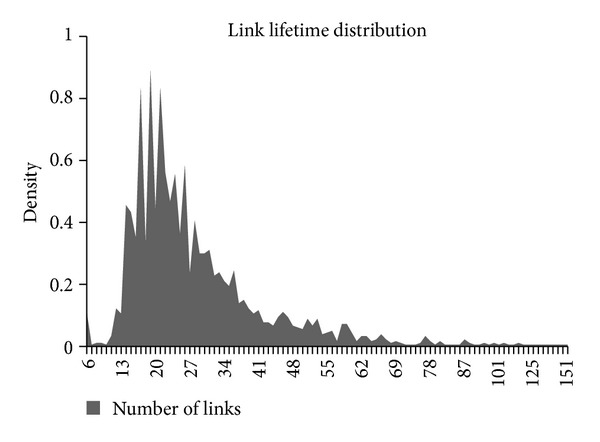
Distribution of link durations in a Random Waypoint scenario.

Also we conducted our own simulation to validate this hypothesis, as it's shown in (Figure 5).

**Definition 1 (Random variable of link lifetime)**

We considered the set *𝕍* of nodes forming the network as population whose elements possess a measurable character which is the realization of a random variable (link lifetime) that follows a normal or Gaussian distribution (*m*; *σ* ^2^)*X*. We assume a finite population and |*𝕍*| ≫ *N*_1_(*V*_*i*_):

(1) $$\begin{array}{c} f\left(x\right)=\frac{1}{\sigma \sqrt{2\pi }}e^{-(1/2){\left((x-m)/\sigma \right)}^{2}}. \end{array}$$

Each node *V*_*i*_ samples a random set of *n* nodes (links); then we obtain the following series of values *x*_*i*,1_, *x*_*i*,2_, … *x*_*i*,*n*_.

**Figure 6:**
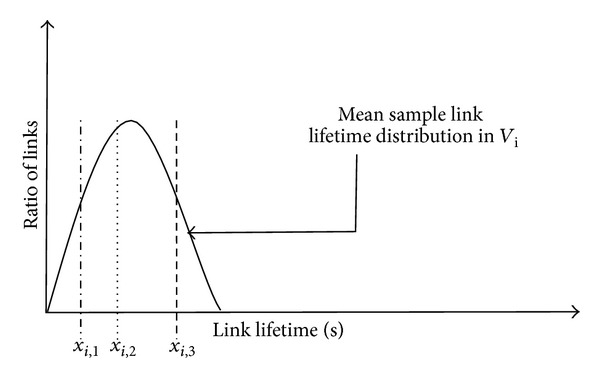
Calculation of remaining link lifetime probability.

The completions of the law *X* as in Figure 6 is given by

(2) $$\begin{array}{c} P\left(x\geq x_{i,j}\right)=\frac{1}{\sigma \sqrt{2\pi }}\int_{x_{i,j}}^{+\infty }e^{-(1/2){\left((x-m)/\sigma \right)}^{2}}dx. \end{array}$$

### 3.2. 2-Hop Connectivity Ratio

Assuming that every node *y*∈*N*_1_(*V*_*i*_) we define a ratio of reachability such as

(3) $$\begin{array}{c} r\left(y\right)=\frac{\left|N_{2}\left(V_{i}\right)\cap N_{1}\left(y\right)\right|}{N_{2}\left(V_{i}\right)}. \end{array}$$

### 3.3. New Decision Process in Multipoint Relay Selection Procedure

Our proposal is to calculate the reliability of a candidate node *V*_*j*_ to be in the MPR set of *V*_*i*_, by being aware of the connectivity and the link lifetime mean emanating from that node to its neighbour. Consider the following:

(4) ℛ(Vj)=  |N2(Vi)∩N1(Vj)|N1(Vi)  ×1σ2π∫xi,j+∞e−(1/2)((x−m)/σ)2dx.

See Procedure 1.

## 4. Simulations Environments

Our proposal is experienced under network simulator NS2 [14] (Network Simulator) 2.35 version in which we have integrated a standard version of OLSR (UM-OLSR-0.8.8 [15, 16]), which is developed by MASIMUM (MANET Simulation and Implementation at the University of Murcia).

Our simulation parameters are as follow. For all simulations, our network is consisted of a maximum number of mobile nodes (70) whose radio scoop is 100 m, moving in an area of 1000 × 1000 m². Each node moves according to the RWP (Random Waypoint) mobility model [17] with pause time fixed to 0 second and maximum speed that varies between 5 and 30 meter/second with step of 5. The scenario that defines the nodes movement is regenerated at the beginning of each simulation. To generate traffic in the network, in each simulation, 1/5 of nodes are randomly selected to be a source of CBR (Constant Bit Rate) traffic. And these selected nodes use UDP (User Datagram Protocol) connections to send Packets with 1024 bytes of size such that one packet every 2.5 second is sent.  Table 1 summarizes all the parameters used during simulations.

## 5. Results and Discussions

In the simulations work set all the possible attributes of network could be highlighted, such as node mobility, network load, the node density, and the number of connections.

In our context we care about link lifetime in mobile environment; indeed we vary the mobility of nodes: we start with a mobility scenario in which the nodes have a low velocity of 5 m/s (18 km/s). We then increase the node velocity up to 25 m/s (90 km/h). Our intention is to investigate the behavior of protocols in networks with varied mobility. We keep a constant data rate of 10 packets/s (40.960 kbps) and a constant number of connections.

Hereafter we compare the resulting performance of our proposal referred to as correlated lifetime and connectivity estimator (CLCE) and the standard OLSR referred as OLSR-STD.

**Figure 7:**
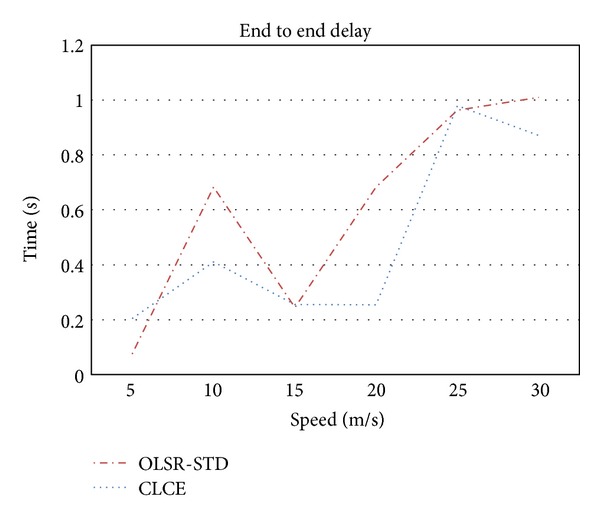
End to end delay comparison.

In Figure 7 we observe that, at the high speed, the performance of end to end delay (elapsed time from sending to reception) of both protocols decreases. However the decreased performance is large, mainly due to link breakage, but it still makes the comparison meaningful.

Generally CLCE presents 16% lower delay the OLSR-STD, especially when velocity reaches higher values; this can be explained by the efficiency that CLCE has in selecting MPR which has more probability to be in the direct neighbourhood. In this way the routes continue to be valid and packet retransmission is decreased. As a consequence end to end delay is lower.

## 6. Conclusions

The key idea of this paper was to apply new link reliability estimation model to the multipoint relays selection and indirectly to routes calculation in the context of a proactive routing protocol, in order to guarantee reliable data transmission. Based on an updated estimation model of link lifetime correlated with a connectivity ratio by this proposal we have considerably optimized the end to end delay from sending to reception. Indeed this new approach could be subject of many application, where delay of packets delivery is critical, namely, in aerospace domain.

## Figures and Tables

**Procedure 1:**
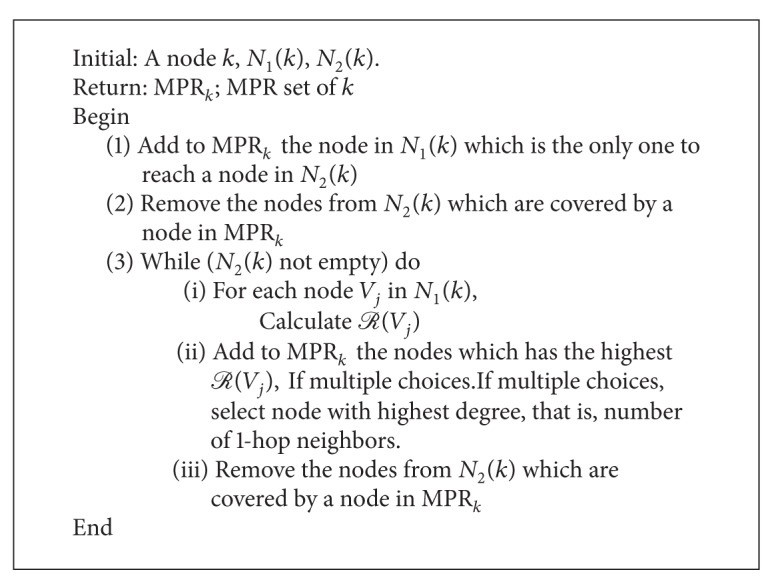
Optimized procedure of MPR selection.

**Table 1:** Simulation parameters.

Simulation environment	Option and parameter
Flat size	1000 m × 1000 m
Max number of nodes	70 nodes
Radio scoop	250 m
MAC layer	IEEE.802.11.peer to peer mode
Transport layer	User Datagram Protocol (UDP)
Traffic model used	CBR
Package size	1024 bytes
Rate	0.4
The number of connections	1/5 of the number of nodes
Mobility model	RWP (Random Waypoint)
Pause time	0 second
Maximum speed of nodes	5, 10, 15, 20, and 30 m/s
Simulation time	300 sec

## References

[1] Nguyen D, Minet P Analysis of MPR selection in the OLSR protocol.

[2] Yamada K, Itokawa T, Kitasuka T, Aritsugi M Cooperative MPR selection to reduce topology control packets in OLSR.

[3] Gomez C, Garcia D, Paradells J Improving performance of a real ad-hoc network by tuning OLSR parameters.

[4] Wei W, Cao J A multi-metric QoS routing method for ad hoc network.

[5] Cervera G, Barbeau M, Garcia-Alfaro J, Kranakis E Mitigation of topology control traffic attacks in OLSR networks.

[6] Ahn JH, Lee T-J A multipoint relay selection method for reliable broadcast in ad hoc networks.

[7] Guo Z, Malakooti S, Sheikh S, Al-Najjar C, Malakooti B (2011). Multi-objective OLSR for proactive routing in MANET with delay, energy, and link lifetime predictions. *Applied Mathematical Modelling*.

[8] Toutouh J, Garcia-Nieto J, Alba E (2012). Intelligent OLSR routing protocol optimization for VANETs. *IEEE Transactions on Vehicular Technology*.

[9] Kim K-I, Kim S-H (2006). Effectiveness of reliable routing protocols in mobile ad hoc networks. *Wireless Personal Communications*.

[10] Loh P, Ma J, Jin H, Yang L, Tsai J (2006). A scalable, efficient and reliable routing protocol for wireless sensor networks. *Ubiquitous Intelligence and Computing*.

[11] Jiang S (2004). An enhanced prediction-based link availability estimation for MANETs. *IEEE Transactions on Communications*.

[12] Jiang S, He D, Rao J (2005). A prediction-based link availability estimation for routing metrics in MANETs. *IEEE/ACM Transactions on Networking*.

[13] Gerharz M, Waal Cd, Frank M, Martini P Link stability in mobile wireless Ad Hoc networks.

[14] Alwan H, Agarwal A Multi-objective reliable multipath routing for wireless sensor networks.

[15] Gomes RL, Moreira WA, Ferreira JJH, Abele AJG (2010). LatinCon14: providing QoE and QoS in wireless mesh networks through dynamic choice of routing metrics. *IEEE Latin America Transactions*.

[16] Koloniari G, Pitoura E (2012). A game-theoretic approach to the formation of clustered overlay networks. *IEEE Transactions on Parallel and Distributed Systems*.

[17] Mahjoub D, El-Rewini H Adaptive constraint-based multi-objective routing for wireless sensor networks.

